# Musashi mediates translational repression of the *Drosophila* hypoxia inducible factor

**DOI:** 10.1093/nar/gkw372

**Published:** 2016-05-03

**Authors:** Agustina P. Bertolin, Maximiliano J. Katz, Masato Yano, Berta Pozzi, Julieta M. Acevedo, Dalmiro Blanco-Obregón, Lautaro Gándara, Eleonora Sorianello, Hiroshi Kanda, Hideyuki Okano, Anabella Srebrow, Pablo Wappner

**Affiliations:** 1 Instituto Leloir, Patricias Argentinas 435, Buenos Aires (1405), Argentina; 2 Division of Neurobiology and Anatomy, Graduate School of Medical and Dental Sciences, Niigata University, 1–757, Asahimachidori, Chuo-ku, Niigata, Niigata 951–8510, Japan; 3 Instituto de Fisiología, Biología Molecular y Neurociencias (IFIBYNE, UBA-CONICET), Ciudad Universitaria, Pabellón 2, Buenos Aires (C1428EHA), Argentina; 4 Departamento de Fisiología, Biología Molecular y Celular, Facultad de Ciencias Exactas y Naturales, Universidad de Buenos Aires, Ciudad Universitaria, Pabellón 2, Buenos Aires (C1428EHA), Argentina; 5 Department of Physiology, Keio University School of Medicine, 35 Shinanomachi, Shinjuku-ku, Tokyo 160–8582, Japan

## Abstract

Adaptation to hypoxia depends on a conserved α/β heterodimeric transcription factor called Hypoxia Inducible Factor (HIF), whose α-subunit is regulated by oxygen through different concurrent mechanisms. In this study, we have identified the RNA binding protein dMusashi, as a negative regulator of the fly HIF homologue Sima. Genetic interaction assays suggested that dMusashi participates of the HIF pathway, and molecular studies carried out in *Drosophila* cell cultures showed that dMusashi recognizes a Musashi Binding Element in the 3′ UTR of the HIFα transcript, thereby mediating its translational repression in normoxia. In hypoxic conditions dMusashi is downregulated, lifting HIFα repression and contributing to trigger HIF-dependent gene expression. Analysis performed in mouse brains revealed that murine Msi1 protein physically interacts with HIF-1α transcript, suggesting that the regulation of HIF by Msi might be conserved in mammalian systems. Thus, Musashi is a novel regulator of HIF that inhibits responses to hypoxia specifically when oxygen is available.

## INTRODUCTION

Animals can adapt to variations of oxygen levels by modifying their transcription profile. Oxygen-dependent gene expression is regulated mostly by the Hypoxia Inducible Factor (HIF), an evolutionary conserved heterodimeric transcription factor, whose α and β-subunits belong to the basic-Helix-Loop-Helix-PAS (bHLH-PAS) protein family (1). While the HIFβ subunit is constitutively expressed, HIFα expression is regulated primarily at the level of protein stability (2). HIFα is rapidly degraded in normoxia and stabilized in hypoxia, being its degradation dependent on the hydroxylation of key prolyl residues localized in the HIFα oxygen-dependent degradation domain (3,4). Hydroxylation of these prolines is mediated by specific prolyl-4-hydroxylases, termed PHDs, that utilise molecular oxygen as a co-substrate for catalysis, and are hence considered oxygen sensors (5,6). The bHLH-PAS proteins Similar (Sima) and Tango (Tgo) are respectively the *Drosophila* HIFα and HIFβ homologs (7), while the *fatiga* gene encodes the *Drosophila* PHD isoforms that control Sima stability in an oxygen dependent manner (8,9). The *Drosophila* HIF system has been shown to control adaptation to hypoxia *in vivo* through mechanisms identical to those operating in mammalian systems (10).

The Musashi (Msi) family of RNA binding proteins is an evolutionarily conserved group of proteins that regulate translation of target mRNAs by binding to consensus sequences, termed Musashi Binding Elements (MBEs), at their 3′ untranslated region (3′ UTR) (11–15). Musashi proteins have clear roles in stem cell maintenance and cell fate determination across the metazoan lineage (16,17). Two Msi paralogs, Msi1 and Msi2, are present in vertebrate species, and a few of their mRNA targets have been so far identified (17). These include the Notch inhibitor Numb (18), the cell cycle regulator CDKN1A/p21 (19), the neural microtubule-associated protein Doublecortin (20), the multidomain tumor suppressor protein Adenomatous Polyposis Coli -APC- (21), the Notch ligand Jagged1 (15), the phosphatase PTEN (22), the integral membrane protein Tetraspanin 3 (23) and the meiotic regulator c-mos in *Xenopus laevis* (24). In *Drosophila melanogaster*, a single *musashi* orthologous gene occurs (*dmsi*) which is known to mediate translational repression of the transcription factor Tramtrack69 (Ttk69), thereby controlling asymmetric cell divisions during adult sensory organ differentiation and photoreceptor differentiation during eye development (11,25,26). dMsi is also required for male germ line stem cell maintenance, although the mRNA target in this context is unknown (27).

In this study we show that dMsi is a novel inhibitor of HIF-dependent responses to hypoxia in *Drosophila*. dMsi recognizes a MBE within the 3′ UTR of *sima* mRNA and mediates its translational repression in normoxic conditions. dMsi is downregulated in hypoxia, lifting Sima repression and contributing to trigger HIF-dependent gene expression. We provide evidence that HIF regulation by Msi might be conserved in mammals.

## MATERIALS AND METHODS

### Identification of Musashi binding elements

To identify RNA regulatory motifs in *sima* or HIFα 3′ UTRs, we used the computational platform RegRNA2.0 (http://regrna2.mbc.nctu.edu.tw/index.html). Several Musashi Binding Elements were inferred through this analysis, and we focused on those conserved between species.

### Fly strains

Flies were reared on a cornmeal-yeast-sucrose medium at 25°C. All the strains used in this study have been previously described. These fly lines were: HRE-LacZ (7), *msi*^1^ (25), *msi*^Df(3R)6203^ (Bloomington stock number 7682), *fga*^9^ and *sima*^07607^ (8), dSRF-Gal4 (28). The following stocks were from the Vienna *Drosophila* RNAi Center: UAS-msi RNAi (VDRC #44895), UAS-sima RNAi (VDRC #106504).

### Cell culture

Semi-adherent Schneider (S2R+) *Drosophila* cells were maintained in Schneider *Drosophila* medium (Sigma) supplemented with Penicillin (50 U/ml, Invitrogen), Streptomycin (50 ug/ml, Invitrogen) and 10% FBS (Invitrogen) at 25°C in 25 cm^2^ T-flasks (Greiner). Synthesis of dsRNA and RNA interference treatments in S2R+ cells were performed as previously described (29).

### Plasmids, transfection and Luciferase assays

For transient transfection experiments in S2R+ cells, we employed previously characterized vectors: pAC-LacZ, HRE-LucFF, pAC-LucRen (30) and pAC-Msi (11). All vectors generated in this manuscript employ the copper-inducible pMT/V5-His plasmid (Invitrogen) as the backbone vector. To obtain pMT-Luciferase *Renilla* (pMT-LucRen), LucRen coding sequence from pRL-SV40 vector (Promega) was directionally cloned into pMT/V5-His using HindIII/XbaI. pMT-Luciferase Firefly reporter construct (pMT-LucFF) was obtained by subcloning the coding sequence of LucFF from pGL3 vector (Promega) into EcoRI/XbaI sites of pMT/V5-His. All 3′ UTRs used here were obtained by PCR of cDNA obtained from *Drosophila* embryos and subsequently cloned into the XbaI/ApaI restriction sites of pMT-LucFF. The employed primers are as follows,

3′ UTR *adh*
Fw: 5′-GCTCTAGAGAAGTGATACTCCCAAAAAA-3′
Rv: 5′-GCCATTGGGCCCATCATAGGAAAATGAATTGC-3′
3′ UTR *ttk*69
Fw: 5′-GCTCTAGATCTCTGGGCACCTCACACCAAG-3′
Rv: 5′-GCCATTGGGCCCGAGTGTTTTTTGCATTGTGTATTT-3′
3′ UTR *sima*
Fw: 5′-GCTCTAGAATTACCAGTACCTTAGCATGCA-3′
Rv: 5′-GCCATTGGGCCCCAAAAACTTTTTTTCTCGTCACAGC-3′

The point mutations in the MBE of *sima* 3′ UTR (3′ UTR sima MBE^mut^) were introduced by nested PCR with the additional primers:

Fw: 5′-CACACTTGAATAGTTTTCTTCCCATGTTAACTGCC-3′
Rv: 5′-GGCAGTTAACATGGGAAGAAAACTATTCAAGTGTG-3′

For transfection experiments, 350.000 cells per well were plated in 24-well plate (Grenier) and 0.3 μg of total plasmid DNA were transfected employing Effectene transfection reagent (Qiagen). All pMT-LucFF-3′ UTR constructs were co-transfected (1:1) with a *Renilla* luciferase plasmid (pMT-LucRen) to normalize transfection efficiency. Expression of all pMT-Luc reporters was induced 24h after transfection by addition of 0.7 mM CuSO_4_ for 7 h. Luciferase activity was measured by using the Dual-Glo Luciferase Assay System (Promega) following the instructions of the manufacturer and measured in a Veritas Microplate Luminometer (Turner BioSystems).

### Reverse transcription and qPCR (RT-qPCR)

Total RNA from S2R+ cells and fly embryos exposed to different treatments was isolated using 500 μl of Trizol reagent (Invitrogen). One μg of total RNA, measured with a *NanoDrop* 1000 spectrophotometer (Thermo Scientific), was used as template for complementary DNA (cDNA) synthesis, using SuperScript III First Strand Synthesis System for RT-PCR (Invitrogen). Quantitative PCR reactions were conducted employing a 1/30 dilution of cDNA sample, SYBRGreen, ROX reference dye and Taq DNA polymerase (Invitrogen) in a Mx3005P real time PCR device (Stratagene). The annealing temperature was 60°C and the elongation time at 72°C was 60 s. Relative mRNA abundances were estimated employing internal standard curves with a PCR efficiency of 100 ± 10% for each set of primer in each experiment. The MxPro qPCR software was used to analyze the data. The *Ribosome protein Large 29* (*RpL29*) gene was used as normalizer. The primers utilized were:

firefly luciferase:
Fw: 5′-CATAGAACTGCCTGCGTGAG-3′ / Rv: 5′-ACCGTGATGGAATGGAACAA-3′
*Renilla* luciferase:
Fw: 5′-AAGTTCGTCGTCCAACATTATC / Rv: 5′-GGCACCTTCAACAATAGCATT-3′
*rpl29*:
Fw: 5′-GAACAAGAAGGCCCATCGTA / Rv: 5′-AGTAAACAGGCTTTGGCTTGC-3′
*lactate-dehydrogrenase (ldh)*:
Fw: 5′-GTGTGACATCCGTGGTCAAG / Rv: 5′-CTACGATCCGTGGCATCTTT-3′
*fgaB*:
Fw: 5′-CACCCTTTCTCTGCACAACA / Rv: 5′-TGTCCAAAAGTTCCCGAAAG-3′
*spermine oxidase*:
Fw: 5′-GCATGGTTGGAGGATGTCTT / Rv: 5′-TCTGGGATTTTCCACCTCAG-3′
*sequoia*:
Fw: 5′-TCGCAGTACACCTTCACGAC / Rv: 5′-AGCAGCTCGTTCTTCAGCTC-3′
*sima*:
Fw: 5′-AGCCCAATCTGCCGCCAACC / Rv: 5′-TGGAGGCCAGGTGGTGGGAC-3′

### SDS-PAGE and immunoblotting

Protein extracts from S2R+ cells or embryos (stage E14 to stage E17) were prepared in RIPA buffer (50 mM Tris–HCl pH 7.5, 150 mM NaCl, 0.1% (v/v) SDS, 0.5% (w/v) sodium deoxycholate and 1% Triton X-100) with the addition of proteinase inhibitory cocktail (Invitrogen) and kept at 4°C. 25–50 μg of total extracts were loaded on a 6–11% polyacrylamide gel, subjected to electrophoresis and then blotted onto nitrocellulose membrane (Bio-Rad). Thereafter, membranes were blocked for 1 h at room temperature with 5% nonfat milk or BSA in TBS with 0.1% Tween 20 (TBS-T) and incubated overnight with rabbit anti-Sima (29); rat anti-d*Msi* 3A5 (26) or mouse anti-*tubulin* (Invitrogen) in 5% nonfat milk in TBS-T. The secondary antibodies used were HRP conjugated (1/5000, Jackson ImmunoResearch). Immunoblots were developed with the ECL prime detection reagent (Amersham).

### β-Galactosidase activity

For X-gal stainings, late-stage embryos were dechorionated in bleach for 1 min, incubated with heptane for 5 min, fixed 20 min at room temperature in glutaraldehyde 0,5% in PBS and washed in PT (0.1% Triton X-100 in PBS). Tissues were incubated in 500 μl of staining solution (5 mM K_4_Fe^2+^, 5 mM K_4_Fe^3+^ and 0.2% of 5-bromo-4 chloro-3 indolyl β-d-galactopyranoside in PT) at 37°C and the colorimetric reaction was monitored. Reactions were stopped by several washes with PT and recorded with a Nomarski Olympus BX-60 microscope.

### RNA Immunoprecipitation (RIP)

S2R+ cells were harvested and lysed in the following extraction buffer (50 mM Tris–HCl pH 7.5, 1% (v/v) NP-40, 0.5% (w/v) sodium deoxycholate, 0.05% (w/v) SDS, 1 mM EDTA, 150 mM NaCl) containing complete protease inhibitor cocktail and RNasin (Promega). Extracts were sonicated with a Bioruptor at high amplitude with three 30-s bursts and insoluble material was precipitated. Supernatant was precleared with GammaBind G sepharose beads (GE Healthcare) for 30 min at 4°C before addition of anti-dMsi or non-immune IgG for incubation overnight. Complexes were immunoprecipitated with GammaBind G sepharose beads for 1 h and after three washes with the above extraction buffer; RNA was extracted with phenol–chloroform. cDNA was prepared from one-half of the RNA from anti-dMsi or IgG RIPs using 10-mer random primers. For every RNA fragment analyzed, each sample was quantified from three independent RIPs. The cDNA and no–reverse transcription control were analyzed by qPCR with the following primers:

adh
Fw: 5′-*AGATAAATGGGAGCGGCAGG*-3′; Rv: 5′-GTGCAATTCCTCCGCAATCC-3′
ttk69
Fw: 5′-GTTAATCCCGGGTCTGGGTC-3′; Rv: 5′-GATGTTACGGGGAACGGTGT-3′
sima
Fw: 5′-CGAATGGCGAAGGTGAAC-3′; Rv: 5′-CTTGGCTGCTTGGGTTTG-3′

### CLIP-RT-qPCR assay

CLIP-RT-qPCR assays were performed as described previously (31) with modifications. Mouse embryonic brains were UV cross-linked at 254nm (UV-B) with 400 mJ/cm^2^ three times, lysed in PXL buffer; and immunoprecipitated for 2 h at 4°C with 4 μg polyclonal anti-mMsi1 or rabbit normal IgG antibodies bound to Dynabeads Protein G (Invitrogen). IPed lysates were washed with PXL, high salt wash buffer and PNK buffer twice respectively to completely remove indirect protein–protein interactions. To purify proteins directly bound to RNA, the complexes on beads were digested with proteinase K (Roche), followed by RNA isolation after phenol/chloroform extraction. RT reaction was performed as described in iScript cDNA Synthesis kit (BioRad). Quantitative RT-PCR was performed using Thunderbird Syber qPCR mix (Toyobo) on the Step-one-plus Real time PCR system (Life Technologies). CLIP-qrt-PCR enrichments were normalized by quantifying relative levels of *gapdh* mRNA, which is not a target of mMsi1. The following primers were used:

gapdh
Fw: 5′-AGGTCGGTGTGAACGGATTTG-3′; Rv: 5′-TGTAGACCATGTAGTTGAGGTCA-3′
HIF-1α
Fw: 5′-TGGAAGGTATGTGGCATTTATTTGG-3′; Rv: 5′-CAGAGGGACTGTTTTGAGTTGGT-3′
Epas1(HIF-2α)
Fw: 5′-GTGTGACAGTCCCAGGAGAGAAG-3′; Rv: 5′-TAGCGGCAACAGCACACAC-3′
HIF-3α
Fw: 5′-TACCTTATTCACCCCTCTTTGGA-3′; Rv: 5′-AGCCAGGACAATTTTTCCGGT-3′
Dcx
Fw: 5′-TTTCAGGAGCAAAACTCTTCAGG-3′; Rv: 5′-TTCTGTTTGGCAGTGAGAGCA-3′

### Analysis of developmental phenotypes

For pupariation analysis, 50 synchronized first-instar larvae were placed in standard food and the number of pupae was measured every 24 h daily until day 8 after larval eclosion. To evaluate the maximal third-instar larval size, 25 synchronized first-instar larvae were placed in standard food and visually monitored throughout their development two times per day. To measure the maximal size reached, 25 third-instar wandering larvae were imaged per genotype. The volume of each larva was calculated from the area measured from photographs using ImageJ.

### Quantification of tracheal phenotypes

Branching quantification was performed as previously reported (28). Briefly, first-instar larvae were placed in fresh vials, at a density of 25 individuals per vial and let them develop to third instar. Wandering larvae were ether anesthetized and ramifications of terminal cells of the third segment dorsal tracheal branch were counted and photographed using bright-field microscopy.

### Hypoxia treatment

Hypoxia was applied in a Forma Scientific 3131 incubator, by regulating the proportions of oxygen and nitrogen at 25°C.

### Statistical analysis

All the statistical analysis were performed using InfoStat version 2009 (Grupo InfoStat, FCA, Universidad Nacional de Córdoba, Argentina). In all cases normality and variance homogeneity were tested with the Shapiro-Wilk test and Levene's test, respectively. For all graphs, the error bars represent the standard error of the mean (SEM) of at least three independent experiments. The experimental groups with different letters indicate statistically significant differences. *P*<0.05 was considered statistically significant.

## RESULTS

### dMusashi behaves as a negative regulator of HIF/Sima

**Figure 1.**
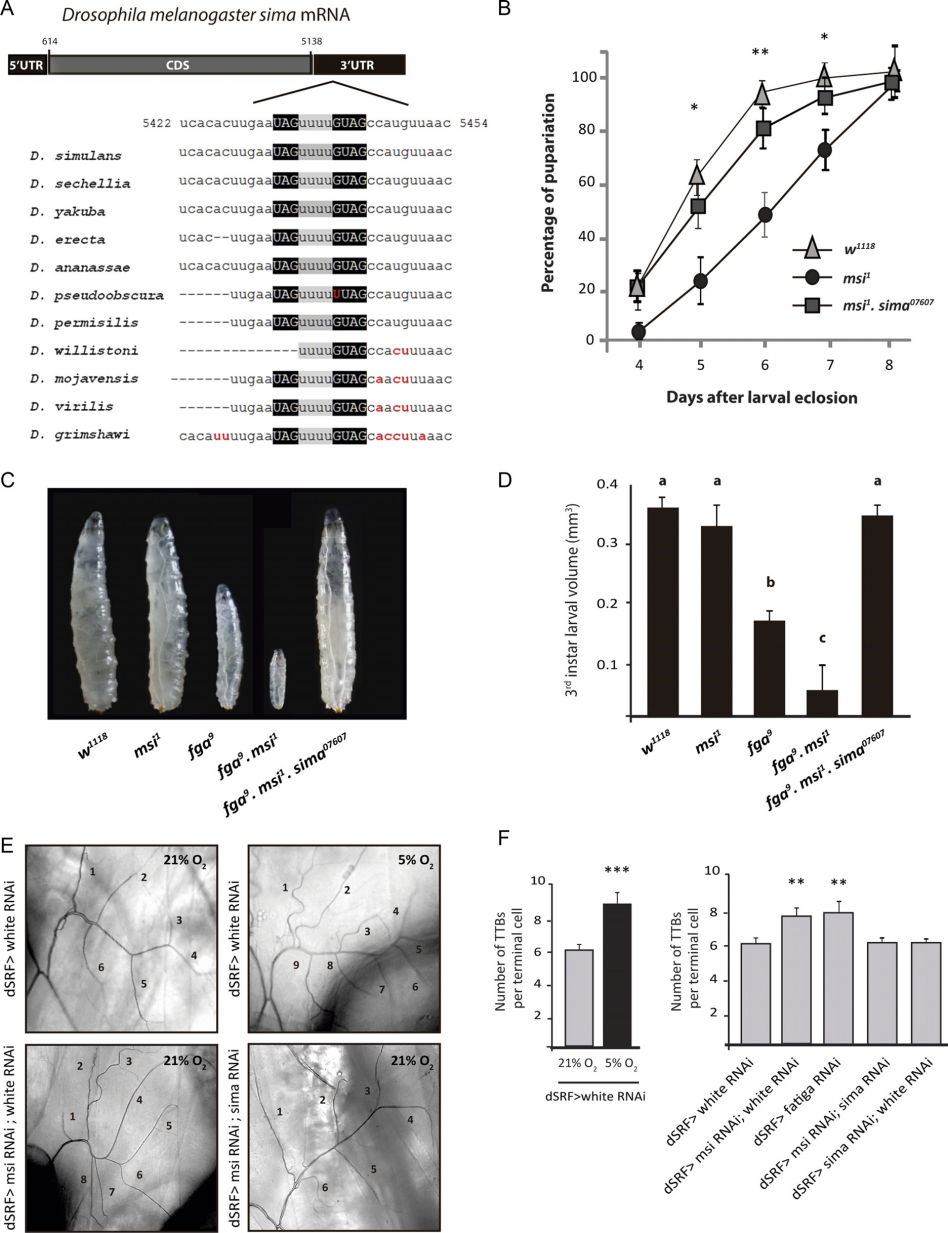
dMusashi loss of function provokes Sima-dependent growth defects and enhances tracheal sprouting. (**A**) Layout of the *Drosophila melanogaster sima* transcript (FBtr0344374). A predicted Musashi binding element (MBE) occurs in the 3′ UTR of all the species analyzed of the *Drosophilidae* family. Minimal binding sequences for Msi (UAG and GUAG) are highlighted in black boxes, while the linker region between them is shown in grey. The nucleotides non-conserved between species are shown in red. (**B**) *musashi* homozygous mutants (*msi^1^*) display prolonged larval development, resulting in delayed pupariation in comparison to their wild type siblings (*w^1118^*), while in *msi^1^, sima^07607^* double homozygotes, normal pupariation timing is restored (error bars represent SEM; *n* = 3 with a minimum of 60 individuals analyzed per genotype at each point of the curve; *P < 0.05 and **P ≤ 0.01; Student's *t* test compared with *msi^1^*). (**C**) Whereas *musashi* (*msi^1^*) homozygous mutants and wild type third instar larvae attain similar size before entering pupariation, *fga^9^* homozygotes are smaller. *fga^9^ msi^1^* double homozygous mutants reach the third larval instar but are remarkably smaller than *fga^9^* homozygotes and never pupariate. In *fga^9^ msi^1^ sima^07607^* triple homozygous third instar larvae, normal growth is rescued. (**D**) Quantification of final body volume of third instar larvae in the experiment depicted in panel C. Error bars represent SEM (*n* = 3 with a minimum of 25 larvae analyzed per genotype in each experiment; different letters indicate statistical differences with a *P* < 0.05 in a one-way ANOVA with a Bonferroni post-hoc test). (**E**) Photographs of the morphology of a terminal cell of the third dorsal branch of a third instar larva in different genotypes or oxygen concentrations are shown. Numbers indicate individual subcellular extensions of more than 1 μm diameter, the ‘‘thick terminal branches’’ (TTBs). Thinner terminal branches may ramify from TTBs but these were not counted. Ramification of terminal cells is enhanced in hypoxic (5% O_2_ 16 h) wild type control larvae (*dSRF*> *white RNAi*), as well as in individuals with dMsi loss of function in these cells (*dSRF* > *msiRNAi*; *white RNAi*) maintained in normoxia. Sima knockdown, along with dMsi silencing, leads to rescue of the normal ramification pattern. (**F**) Quantification of the results shown in panel E. Error bars represent SEM (*n* = 3 with a minimum of 25 larvae analyzed per genotype in each experiment; **P ≤ 0.01; ***P ≤ 0.001; Student's *t* test).

Bioinformatic analysis of *sima* 3′ UTR revealed the occurrence of a dMsi binding element (MBE) conserved across species of the *Drosophilidae* family (see Materials and Methods; Figure 1A). This phylogenetic sequence conservation in *sima* 3′ UTR suggests that dMsi plays a role in *Drosophila* HIFα regulation. To begin analyzing this possibility, we performed genetic interaction assays to assess if dMsi contributes to developmental phenotypes that are known to depend of the HIF pathway in *Drosophila* larvae. We have previously reported that hypomorphic mutants of *fatiga* (*fga^9^*), the main negative regulator of Sima, exhibit delayed pupariation and larval growth impairment (8), and that in *fga^9^ sima^07607^*double homozygous larvae normal growth and developmental timing are restored, indicating that an excess of Sima accounts for these developmental phenotypes. To begin exploring if dMsi negatively regulates Sima, we analyzed pupariation timing of *dmusashi* loss-of-function homozygous animals (*msi^1^*). Compared to control siblings, *msi^1^* mutants show a 1–2 days pupariation delay (Figure 1B), which is reverted in *msi^1^ sima^07607^* double homozygotes (Figure 1B), indicating that the effect of dMsi loss of function is due to Sima accumulation. These results suggest that dMsi may be a negative regulator of Sima. To further investigate the possibility that dMsi inhibits Sima, we analyzed genetic interactions between *msi^1^* and *fga^9^* mutants. The final size of *msi^1^* third instar mutant larvae is indistinguishable from that of controls, while the size of *fga^9^* homozygous larvae is clearly reduced (Figure 1C and D and (8)). Noteworthy, *msi^1^ fga^9^* double homozygous third instar larvae fail to pupariate and are remarkably smaller than *fga^9^* single mutants (Figure 1C and D), although they can remain alive for up to 2 weeks. Strikingly, *msi^1^ fga^9^ sima^07607^* triple homozygous larvae undergo pupariation normally, attaining a final size similar to that of wild type controls (Figure 1C and D), suggesting that dMusashi cooperates with Fatiga in inhibiting the Sima pathway.

Tracheal terminal cells of *Drosophila* third instar larvae are plastic and respond to hypoxia by projecting terminal branches in a Sima dependent manner (28), so we investigated if dMsi modulates tracheal sprouting. As we have previously shown, the number of terminal branches with more than 1μm diameter (‘thick terminal branches’, TTBs) of the dorsal branch of the third segment of third instar larvae is a sensitive parameter to quantify terminal tracheal branching after physiological or genetic interventions (Figure 1E and F left panel; (28)). We used a UAS-msi RNAi line that effectively downregulates dMsi protein levels (Supplementary Figure S1), expressed under control of the terminal cell-specific driver *dSRF*-Gal4 in normoxic larvae, and observed a significant increase in the number of TTBs in comparison to controls expressing an unrelated RNAi (Figure 1E and F right panel). To investigate if this tracheal ramification increase depends on Sima, we coexpressed *msi* RNAi along with a *sima* RNAi, and observed a complete reversion of the phenotype, with the number of TTBs being restored to wild type levels (Figure 1E and F right panel). These results indicate that dMusashi depletion can induce Sima-dependent tracheal terminal sprouting, which is a physiological response to hypoxia in *Drosophila*.

### dMusashi controls Sima protein levels

**Figure 2.**
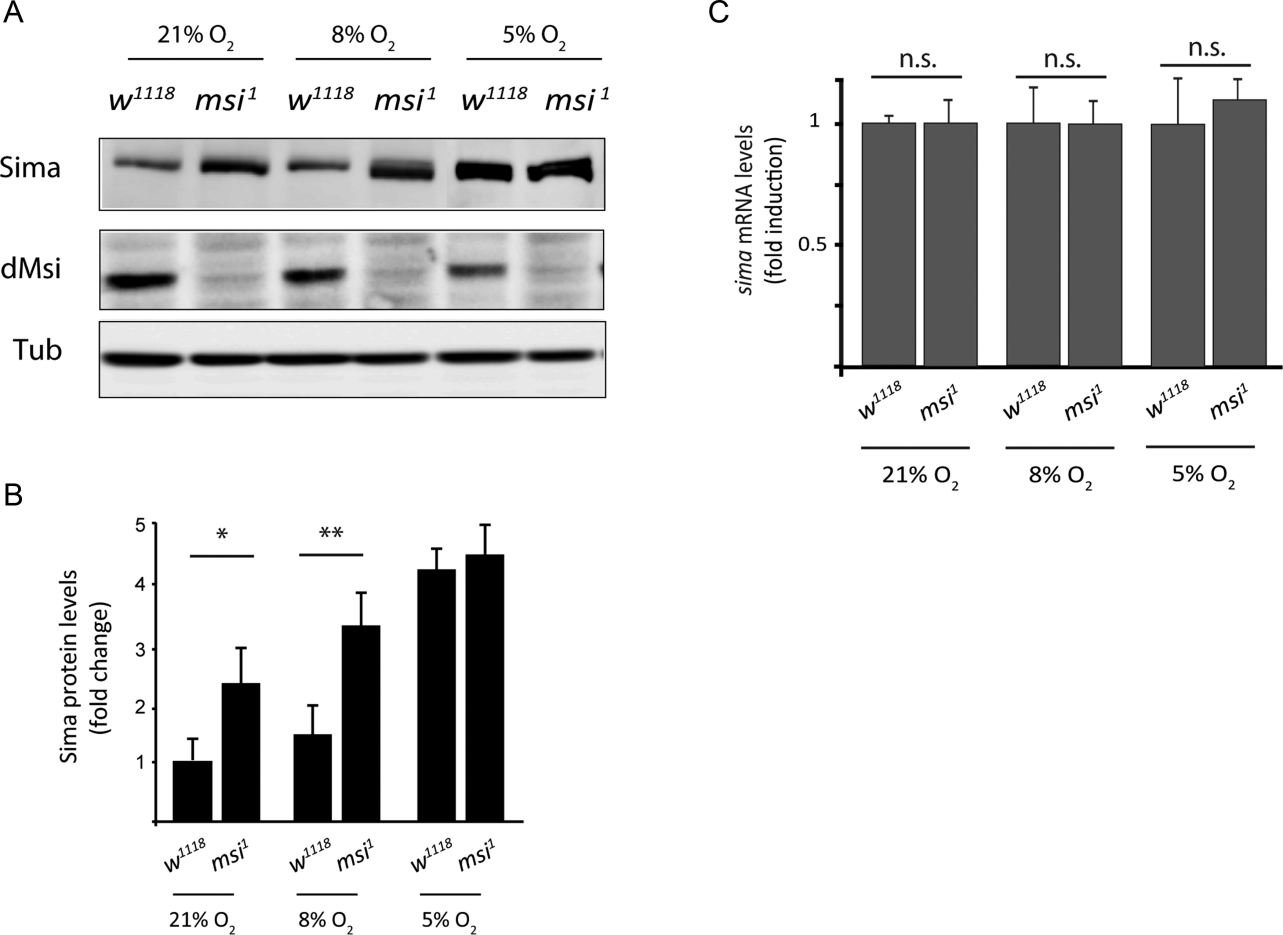
dMusashi downregulates Sima protein levels. (**A**) Western blot showing that Sima protein levels increase in *musashi^1^* homozygous mutant late-stage embryos (*msi^1^*) in comparison to wild type controls (*w^1118^*) at different oxygen concentrations. Sima accumulates in wild type embryos *(w^1118^*) in hypoxia, while in *msi^1^* mutants, Sima accumulates in normoxia or mild hypoxia (8% O_2_ 5 h), but not in strong hypoxia (5% O_2_ 5 h), when compared with wild type controls. Tubulin was used as loading control. (**B**) Quantification of Sima protein levels from the western blot shown in panel A. Error bars represent SEM (*n* = 3, *P < 0.05; **P ≤ 0.01; Student's *t* test). (**C**) *sima* mRNA levels were analyzed by RT-qPCR from embryos in the same conditions as in panels A and B in normoxia or hypoxia. *sima* transcript levels are not significantly different between wild type and mutant individuals in any condition. Rpl29 mRNA was used for normalization. n.s.: values not significantly different, Student's *t*-test.

**Figure 3.**
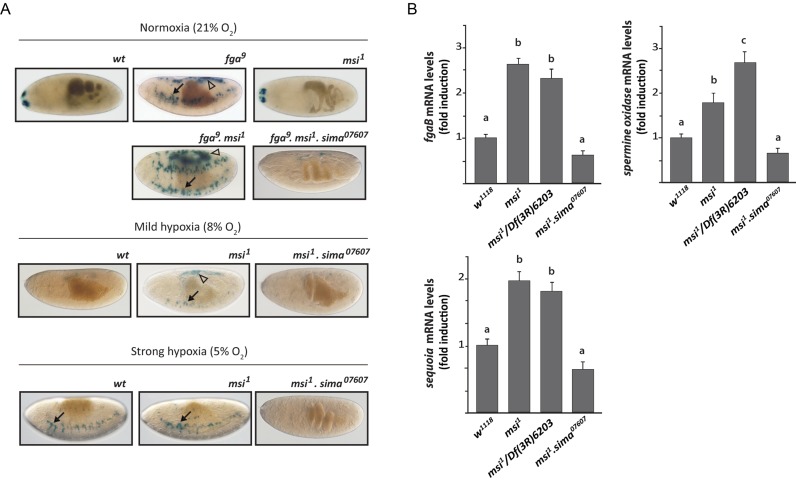
dMusashi downregulates Sima dependent transcription. (**A**) Expression of the Sima-inducible reporter HRE-LacZ in wild type, *fga^9^*or *msi^1^* mutant embryos maintained in normoxia or exposed to two different hypoxic conditions; arrows indicate groups of tracheal cells that express the reporter with maximal sensitivity, and arrowheads indicate groups of dorsal cells where the reporter expression is more variable but occurs consistently when HIF-dependent transcription is especially strong. In wild type embryos, the reporter is only expressed in strong hypoxia (5% O_2_ 5h), while in *fga^9^* mutants expression can be detected even in normoxia (21% O_2_), and in *msi^1^* mutants it is already detectable in mild hypoxia (8% O_2_ 5 h); *fga^9^ msi^1^* double homozygous mutants display overall enhanced expression of the reporter in normoxia in comparison to *fga^9^* single mutants. (**B**) Expression of three Sima target genes was analyzed by RT-qPCR in wild type, *musashi* loss-of-function (*msi^1^* homozygous mutants or larvae with the heteroallelic combination *msi^1^/Df(3R)6203*) or in *msi^1^ sima^07607^*double homozygous mutant third instar larvae maintained in normoxia. In both *msi* loss-of-function backgrounds (*msi^1^* or *msi^1^/Df(3R)6203*), expression of the three Sima target genes, *fgaB, sequoia* and *spermidine oxidase*, is higher than in wild type controls or in *msi^1^ sima^07607^*double mutants. Error bars represent SEM (*n* = 3; different letters indicate statistical differences with a *P* < 0.05 in a one-way ANOVA with a Bonferroni post-hoc test).

To investigate if dMusashi directly controls Sima protein abundance, we performed western blot analysis with anti-Sima antibodies of extracts of *msi^1^* homozygous embryos in comparison to wild type controls in normoxia at 8% O_2_, an hypoxic condition previously shown to trigger mild HIF-dependent responses in *Drosophila* embryos (7), and at 5% O_2_, a condition that induces maximal HIF-dependent transcription in *Drosophila in vivo* (7). As previously reported, Sima accumulates in wild type embryos exposed to hypoxia, and interestingly, Sima protein levels are also increased in *dmsi* mutants, either in normoxia or in mild hypoxia (8% of O_2_) as compared to wild type controls (Figure 2A and B). In strong hypoxic conditions (5% of O_2_), no accumulation of Sima protein is detected in comparison with the wild type (Figure 2A and B). *sima* transcript levels are unaffected in all cases (Figure 2C), suggesting that dMsi downregulates Sima at a post-transcriptional level. Next, we studied if dMsi plays a role in the regulation of HIF-dependent transcription, by analyzing the expression of a hypoxia-inducible transcriptional reporter (HRE-LacZ) in transgenic fly lines (7). As previously reported, HRE-LacZ is silent in wild type embryos in normoxia (21%O_2_) or mild hypoxia (8% O_2_), and induced in strong hypoxic conditions (5% O_2_) (Figure 3A); in *fga* mutant embryos, the reporter is strongly expressed already in normoxia (Figure 3A) (7,8). Interestingly, in *dmsi* null mutants, expression of the reporter can be observed already in mild hypoxia (Figure 3A), suggesting that dMsi negatively regulates Sima dependent transcription. Consistent with this notion, in *msi^1^ fga^9^* double mutants, expression of the reporter in normoxia is stronger than in embryos mutant for *fga* only (Figure 3A). As expected, no expression in any condition is observed in embryos carrying the *sima^07607^* loss-of-function allele in combination with the *msi^1^* mutation or with *msi^1^* and *fga^9^* mutations simultaneously (Figure 3A). To confirm that *dmsi* loss of function enhances Sima dependent transcription, we analyzed in *msi^1^* mutant larvae the expression of three different endogenous genes that are upregulated in hypoxia in a Sima-dependent manner (Figure 3B and Supplementary Figure S2). Increased expression of all three target genes is observed, even in normoxic conditions, both in *msi^1^* homozygous larvae and in larvae heterozygous for *msi^1^* and the *Df(3R)6203* chromosomal deficiency that covers the *dmsi* locus (Figure 3B). Expression of Sima target genes is restored to wild-type normoxic levels in *msi^1^ sima^07607^* double homozygous larvae (Figure 3B), indicating that the enhanced expression of Sima target genes in *dmsi* loss-of-function larvae is due to overaccumulation of Sima. Noteworthy, the extent of upregulation of HIF target gene expression in *msi^1^* mutant normoxic larvae (2–3-fold; Figure 3B), and the extent of their upregulation in wild type larvae exposed to hypoxia (Supplementary Figure S2) is similar. This comparison strengthens the notion that the Sima-dependent transcriptional response to hypoxia is constitutively activated in *msi^1^* mutants. Consistent with the above results, in cultured S2R+ cells treated with *msi* dsRNA, strong enhancement of Sima-dependent transcription is observed in normoxia, while in hypoxia (1% O_2_) this enhancement is milder (Supplementary Figure S3). Altogether, these results indicate that dMsi mediates reduction of Sima protein in normoxic conditions, thereby restricting Sima-dependent transcription.

### dMusashi binds to *sima* mRNA and represses translation

**Figure 4.**
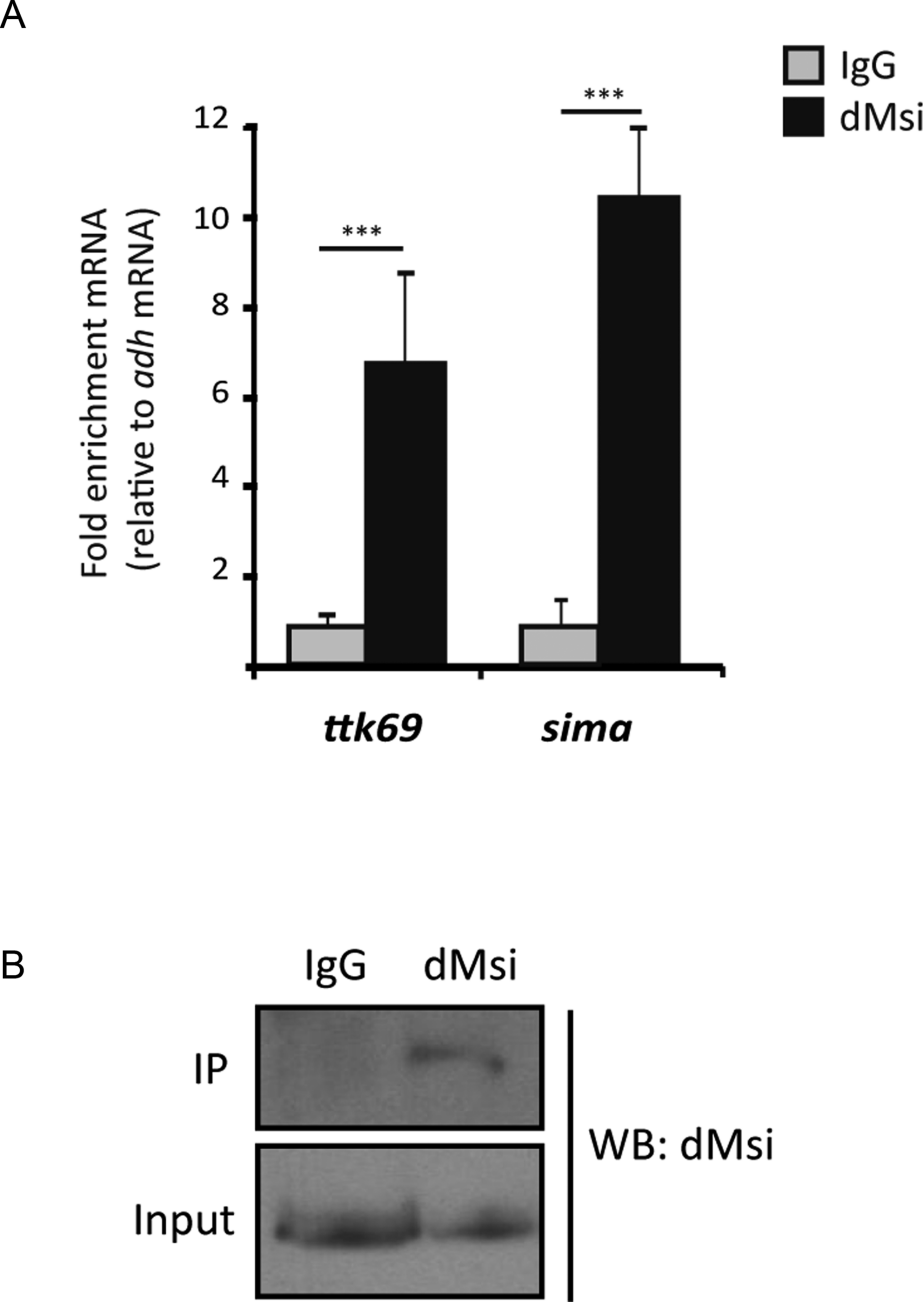
dMusashi binds Sima mRNA. RNA immunoprecipitation (RIP) was performed from extracts of normoxic S2R+ cell cultures using an anti-dMusashi antibody or a pre-immune (IgG) serum. (**A**) Immunoprecipitated *sima* mRNA was quantified by RT-qPCR, along with *ttk69* mRNA (positive control) and *adh* mRNA (that lacks Musashi Binding Elements) for normalization. dMsi binds *sima* mRNA with an efficiency comparable to that of *ttk69* mRNA. (**B**) Western blot showing dMsi protein levels in whole cell extracts (input), and after IP; the protein is immunoprecipitated with the anti-dMsi antibody (dMsi) but not with the pre-immune serum (IgG). Error bars represent SEM (*n* = 3, ***P ≤ 0.001; Student's *t* test).

**Figure 5.**
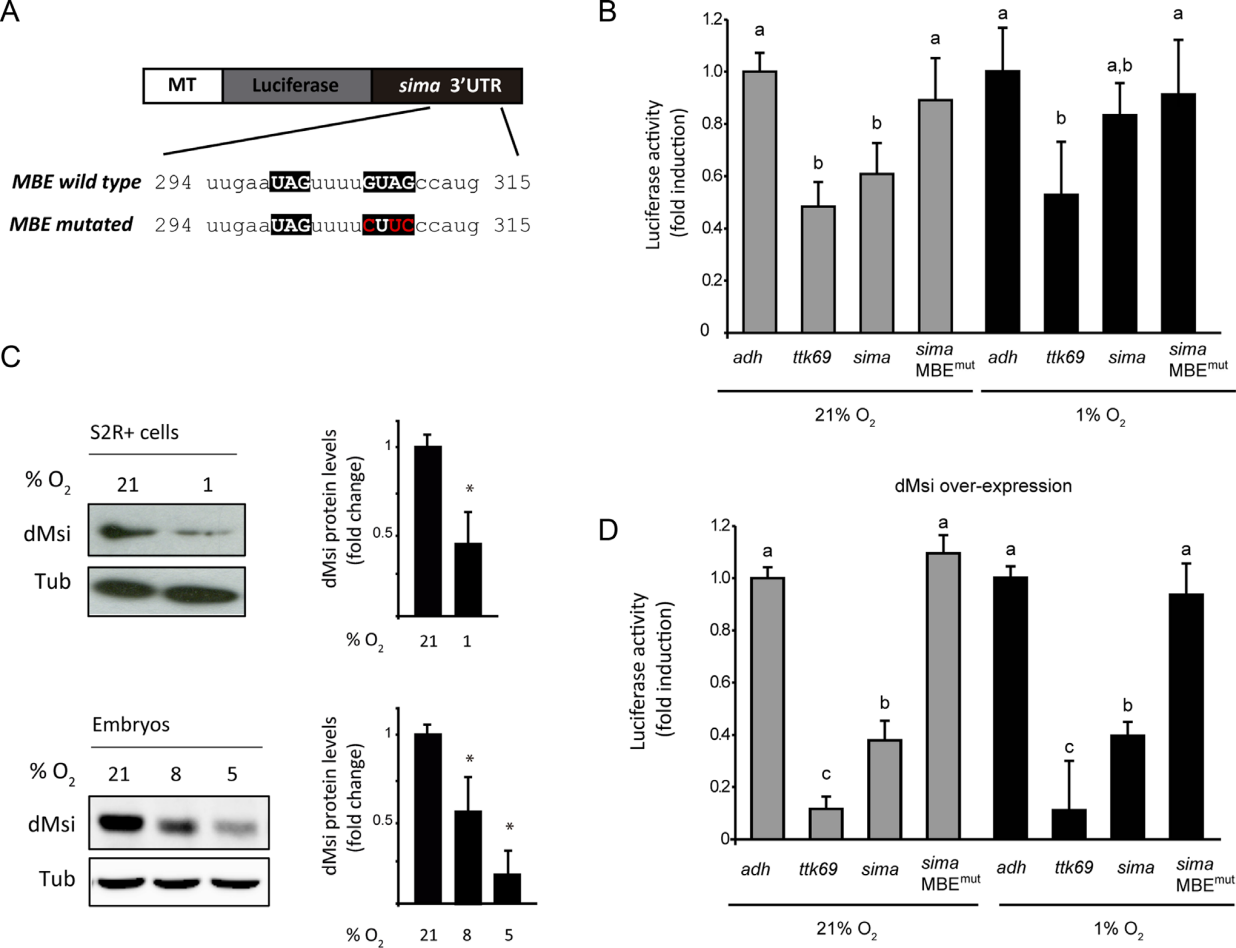
dMusashi represses Sima translation in normoxia. (**A**) Schematic representation of *firefly luciferase* reporter constructs containing wild type or mutagenized versions of the *sima* 3′ UTR. Mutations (shown in red) have been generated within the Musashi Binding Element (MBE) by site-directed mutagenesis. The reporters were transiently transfected in S2R+ cells and expressed under control of a copper-inducible metallothionein promoter (MT). (**B**) S2R+ cells transfected with luciferase reporters containing the *alcohol dehydrogenase* (*adh*) 3′ UTR (negative control), *tramtrack69* (*ttk69*) 3′ UTR (positive control), or *sima* 3′ UTR (either wild type or mutagenized, MBE mut) were maintained in normoxia or exposed to hypoxia (1% O_2_ 20 h), and luciferase activity of cell lysates was measured, using a copper-inducible Renilla luciferase construct for normalization. In normoxic but not in hypoxic cells, *sima* 3′ UTR represses luciferase activity to a similar extent to *ttk69* 3′ UTR; *sima* 3′ UTR mutagenized in the MBE loses completely its repressive activity. (**C**) Western blot analysis of dMsi protein levels in S2R+ cells in normoxia or hypoxia (1% O_2_ 20 h), as well as in embryos maintained in normoxia (21% O_2_) or exposed to hypoxia (8% or 5% O_2_ for 5h). Both in cell culture and *in vivo*, dMsi protein levels are sharply reduced in hypoxia. Tubulin was used as a loading control. Error bars represent SEM (*n* = 3, *P < 0.05; **P ≤ 0.01; Student's *t* test). (**D**) Cells transfected with the same set of reporters as in panel B) were co-transfected with a dMsi overexpression vector (pAc-dMsi) and maintained in normoxia or exposed to hypoxia (1% O_2_ 20 h). dMsi overexpression enhances the repression exerted by *ttk69* or *sima* 3′ UTRs irrespective of oxygen levels (compare with panel B), while the mutagenized version of *sima* 3′ UTR loses its repressive capacity. Luciferase activity is depicted as fold induction respect to the activity of the *adh* 3′ UTR reporter (negative control). In (B) and (D), error bars represent SEM (*n* = 3; different letters indicate statistical differences with a *P* < 0.05 in a one-way ANOVA with a Bonferroni post-hoc test).

As a next step, we sought to explore the mechanism by which dMsi represses Sima. Given the occurrence of a MBE at the 3′ UTR of *sima* mRNA, we analyzed if dMsi protein can bind the *sima* transcript. RNA immunoprecipitation (RIP) analysis with an anti-dMsi antibody indicates specific binding to *sima* mRNA, which occurs with an efficiency comparable to binding of dMsi to *ttk69* mRNA (Figure 4A and B), a previously reported dMsi target. Next, we investigated if the *sima* 3′ UTR, that contains a MBE, can mediate repression of a luciferase reporter in S2R+ cells. In cells maintained in normoxia, *sima* 3′ UTR exerts strong repression of luciferase reporter activity, which is comparable to the repression exerted by the *ttk69* 3′ UTR used as a positive control (Figure 5A and B) (11). The repressive capacity of *sima* 3′ UTR is largely abolished when the MBE is mutagenized (Figure 5A and B), suggesting that repression is mediated by dMsi. Remarkably, the *sima* 3′ UTR is less efficient in repressing luciferase activity in cells exposed to hypoxia (1% O_2_ for 20 h) (Figure 5B), which is consistent with a sharp reduction of dMsi protein levels (Figure 5C) in hypoxia. dMsi transcript levels do not vary in hypoxic conditions, suggesting that dMsi regulation by oxygen is post-transcriptional (Supplementary Figure S4). To get additional evidence that dMsi is responsible for the repression of luciferase activity mediated by *sima* 3′ UTR, we induced dMsi overexpression by cotransfecting cells with an *actin*-dMsi plasmid along with the 3′ UTR luciferase reporters (Supplementary Figure S5). Strong enhancement of the repression of both *sima* 3′ UTR and *ttk69* 3′ UTR luciferase reporters occurs in these cells in normoxia, while again, no repression is observed with the reporter in which the *sima* 3′ UTR was mutagenized at the MBE (Figure 5D). These results confirm that dMsi mediates the repression exerted by *sima* 3′ UTR. Noteworthy, over-expression of artificially high levels of dMsi protein in these cells, overrides the downregulation of dMsi protein levels provoked by the hypoxia treatment (Supplementary Figure S5). Consistent with this, oxygen dependence of *sima* 3′ UTR repressive capacity is lost completely, as repression is equally potent in normoxia and hypoxia (Figure 5D). RT-qPCR of luciferase transcript levels revealed no differences between constructs bearing different 3′ UTRs (Supplementary Figure S6), suggesting that Musashi represses Sima at a translational level, which is consistent with its well-established function as a translational repressor (17).

### The Msi-HIF axis in mammals

**Figure 6.**
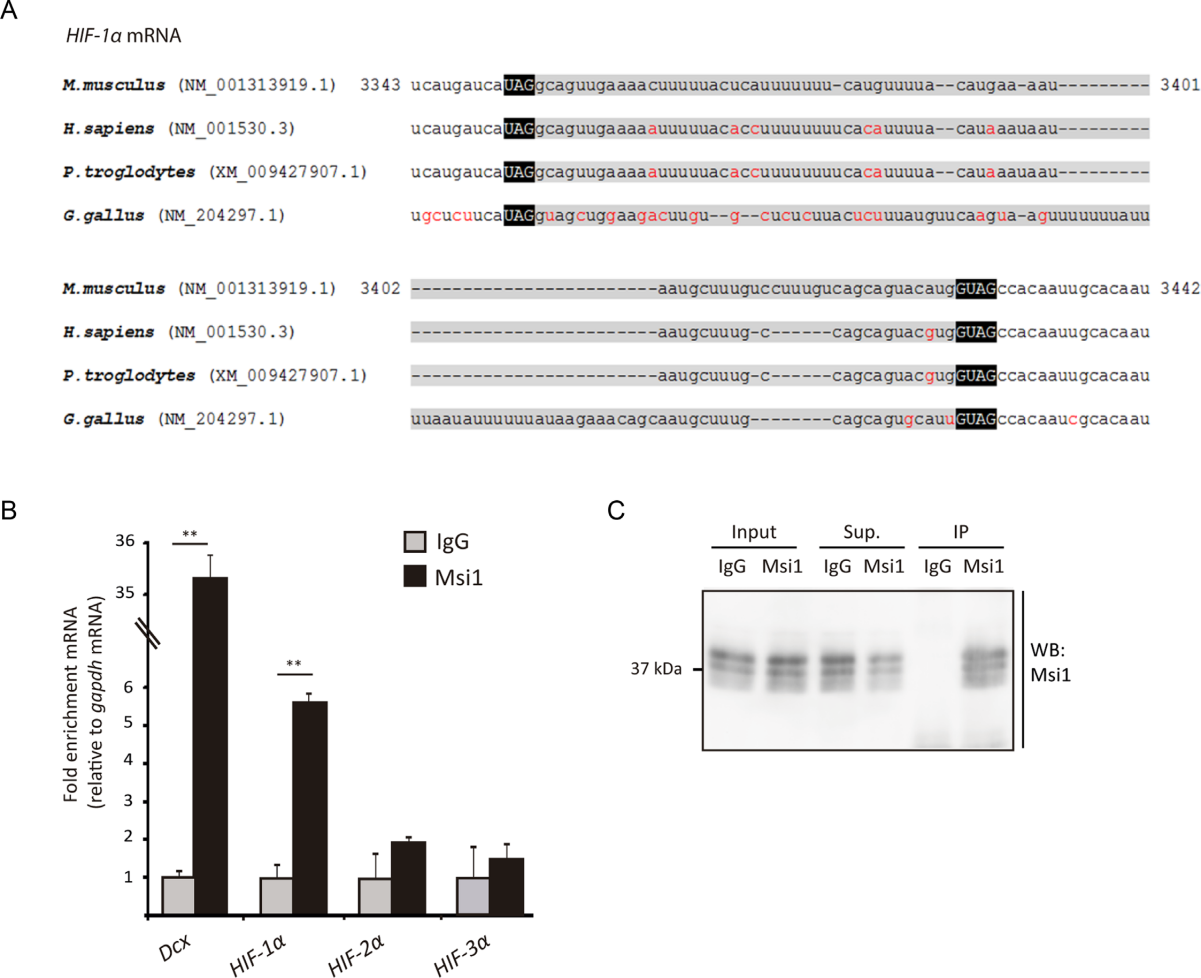
Mammalian Msi1 interacts with HIF-1α mRNA. (**A**) Sequence alignment of HIF-1α 3′ UTRs of vertebrate species is shown. The bipartite Musashi Binding Element (MBE) is highlighted in black boxes. Note that sequence conservation is not limited to the MBE but includes extensive portions of the linker between the two boxes, as well as regions upstream to the 5′ UAG box and the 3′ GUAG box. Nucleotides shown in red color are not conserved between species. (**B**) Murine Msi1 (Msi1) interacts with *HIF-1α* mRNA *in vivo*; CLIP-RT-qPCR assays were performed from extracts of mouse embryonic brains using an anti-Msi1 or control antibody (IgG). Msi1-bound mRNAs was quantified by RT-qPCR, using the *gapdh* transcript for normalization. Msi1 protein interacts with HIF-1α but not with HIF-2α or HIF-3α mRNA (*n* = 3; error bars represent standard deviation). Asterisks indicate *P* < 0.01 by Student's *t* test. (**C**) Western blot showing dMsi protein levels in whole cell extracts (input), and after IP, in the supernatant (Sup.) or in the pellet (IP); the protein can be immunoprecipitated with the anti-dMsi antibody (dMsi) but not with the pre-immune serum (IgG).

Having established that Msi regulates HIF in the *Drosophila* system, we asked whether this regulation might be conserved in mammals. Bioinformatic analysis of mammalian HIFα 3′ UTRs (see Materials and Methods) revealed the occurrence of a putative MBE in HIF-1α 3′ UTR (Figure 6A), but not in the 3′ UTRs of HIF-2α or HIF-3α. Noteworthy, the sequence is evolutionary conserved and conservation is not limited to the UAGx_n_GUAG bipartite Msi-binding consensus sequence (13), but rather, substantial conservation is also observed upstream to the UAG 5′ box and downstream to the 3′ GUAG box, as well as in the linker region (Figure 6A). These observations prompted us to analyze if mammalian Msi1 protein binds HIFα transcripts in a mammalian system. We carried out CLIP-based RT-qPCR assays (31) to analyze possible interactions in mouse embryonic brains (E14.5), using the *doublecortin* (*Dcx*) mRNA as a positive control (20). We performed UV-crosslinking on tissue samples, and prepared an extract (see Materials and Methods), which was subjected to immunoprecipitation with an anti-Msi1 antibody, followed by RT-qPCR analysis of HIF-1α, HIF-2α and HIF-3α transcripts. Brain extracts were prepared in stringent buffer conditions to minimize protein-protein non-covalent interactions and indirect protein-RNA interactions. As shown in Figure 6B and C, Msi1 physically interacts with HIF-1α mRNA but not with HIF-2α or HIF-3α transcripts. These observations suggest that Msi1 might potentially regulate HIF-1α translation in the mouse brain, as we have shown above for the *Drosophila* homologous proteins.

**Figure 7.**
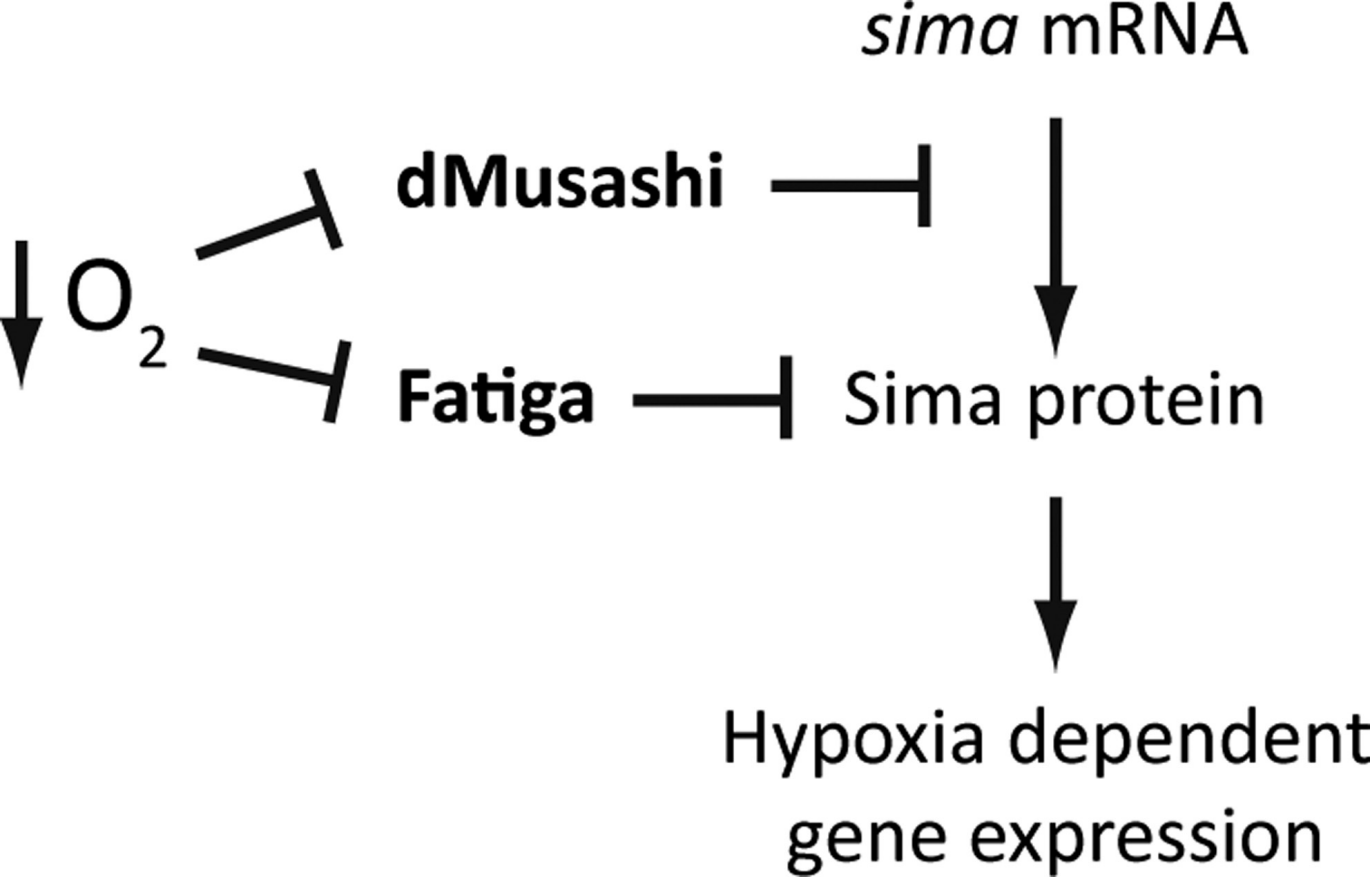
Model for the regulation of Sima by dMusashi. The prolyl-4-hydroxylase Fatiga downregulates Sima protein levels by promoting its proteasomal degradation, while dMusashi represses *sima* mRNA translation by binding a Musashi Binding Element within the 3′ UTR. dMsi protein levels are reduced in hypoxia, allowing Sima dependent transcription.

Thus, we have shown in the *Drosophila* system that dMusashi inhibits HIF dependent transcriptional responses in normoxia by repressing *sima* mRNA translation. In hypoxia, dMusashi is downregulated and *sima* translational repression is lifted, contributing to activation of HIF-dependent transcription and adaptation to hypoxia (Figure 7). We also found that in mouse embryonic brains, Msi1 physically interacts with the HIF-1α transcript suggesting that Msi-HIF functional interactions might be conserved in mammalian systems.

## DISCUSSION

In this work, we have established a role of the RNA binding protein dMusashi in the regulation of mRNA translation of the hypoxia inducible factor alpha subunit (HIFα). The *Drosophila* HIF alpha-subunit Sima is regulated primarily by oxygen-dependent proteasomal degradation (7,8). The Proline 850, localized in the oxygen-dependent degradation domain, is hydroxylated by the prolyl-4-hydroxylase Fatiga and degraded at the 26S proteasome. Sima oxygen-dependent subcellular localization is another important regulatory step: In hypoxia the protein accumulates in the nucleus, while following reoxygenation, Sima is exported very rapidly to the cytoplasm (2-5 min) by the nuclear export receptor CRM1 (32). Sima nuclear export is also dependent on Fatiga-dependent hydroxylation of Proline 850 (33).

The current work led to the identification of the translational repressor dMusashi as a novel regulator of Sima in *Drosophila*, which adds another layer of complexity to the control of transcriptional responses to hypoxia. We have shown both in cell culture and *in vivo* that dMsi is an inhibitor of HIF in normoxia, which operates by binding a Musashi Binding Element (MBE) in the Sima 3′ UTR and inhibits its translation. In hypoxia dMsi levels are strongly reduced, resulting in suppression of the inhibitory effect. Consistent with these observations, in *msi* mutants HIF-dependent transcription and biological outcomes, including sprouting of the tracheal system, are enhanced. We have provided evidence that Msi–dependent regulation of the HIF system might be conserved at least within higher eukaryotes, as Msi1 protein physically interacts with the HIF-1α transcript in embryonic mouse brains. Defining if HIF-1α regulation by Msi1 occurs in mammals is undoubtedly of interest, and requires further research.

Previous studies on mammalian HIF regulation have focused mostly on oxygen-dependent proteasomal degradation of its α-subunit (3–6,34), and transcriptional co-activator recruitment (35–37), while mechanisms supporting transcriptional (38–40) or translational control (41) are less well-defined. Nonetheless, it is clear that multiple mechanisms that mediate HIF translational regulation occur, contributing substantially to the regulation of responses to hypoxia. One of such mechanisms is the switch from CAP-dependent to CAP-independent (internal ribosomal entry site (IRES)-dependent) mRNA translation. In hypoxia, general CAP-dependent translation is largely suppressed, following Target of Rapamycin (TOR) inhibition, and activation of the eukaryotic translation initiation factor 4 binding proteins (4E-BPs), which bind 4E thereby preventing formation of the initiation complex (42,43). In hypoxia, HIF-1α translation becomes dependent on an IRES localized within the 5′ UTR of its mRNA, thereby escaping general translational inhibition (44). Besides its role in the regulation of CAP-dependent translation through 4E-BPs, TOR is required for HIFα translation, and thereby for triggering the hypoxic response. TOR mediates S6 kinase phosphorylation, which is in turn necessary for HIFα translation in hypoxia (30,45–47).

Another layer of oxygen-dependent regulation of HIFα is the one exerted by RNA binding proteins or micro-RNAs that bind its 5′ or 3′ UTRs, controlling mRNA stability or translation (49). HuR is an RNA binding protein that mediates potent enhancement of HIFα mRNA translation in hypoxia in human cervical carcinoma cells (48). Even though the mechanisms by which hypoxia activates HuR function to potentiate HIFα mRNA translation are so far unclear, it is known that they involve its binding to U or AU-rich sequences at the 5′ UTR. While HuR operates on HIFα 5′ UTR, another RNA binding protein, the polypyrimidine tract-binding protein (PTB) binds its 3′ UTR enhancing translation as well (48). The ability of PTB to enhance HIFα translation depends on HuR, supporting the notion that PTB and HuR cooperate with each other to promote this enhancement (48,49). The T-cell intracellular antigen-1 (TIA-1) and its related protein TIAR have also been reported to regulate HIFα translation. TIA-1 and TIAR are upregulated in hypoxia in a model of rat brain ischemia (50), and form stress granules mediating HIF repression (51). The effect is conveyed by AU-rich elements present at the 3′ UTR of HIF-1α mRNA (51). It remains to be established if HuR, PTB or TIA-1/TIAR homologs are involved in HIF regulation in the *Drosophila* system, and if so, to what extent they functionally interact with dMsi.

The potential biological implications of HIF regulation by the translational repressor Musashi, reported in this study, are appealing. Msi is highly expressed in several types of tumours, being required for maintenance of the undifferentiated state in aggressive leukemias (52–54); in most types of cancer its expression correlates with poor prognosis (15,55–58). Msi in addition participates in asymmetric cell divisions, epithelial-mesenchymal transitions (EMTs) and is a key determinant of stem cell maintenance in diverse organs and cellular contexts, including cancer stem cells (15,17). Remarkably, the biological processes regulated by HIF overlap with those controlled by Musashi: HIF promotes EMT and metastatic phenotypes in human cells and in mice (59); HIF plays a role in stem cell homeostasis in the hematopoietic lineage (60); and finally, HIF plays a pivotal role in tumorigenesis (61,62). Further research is required to determine if the functional relationship of HIF and Musashi described in this work plays a role in one or more of the above biological processes in which the two proteins suggestively overlap.

## Supplementary Material

SUPPLEMENTARY DATA
