# Supplementary material for: Musashi mediates translational repression of the Drosophila hypoxia inducible factor
Source: Nucleic Acids Res. 2016 May 3;44(16):7555–67. doi: 10.1093/nar/gkw372 (PMC5027473; doi:10.1093/nar/gkw372)
Supplement: SUPPLEMENTARY DATA [file supp_44_16_7555__index.html]

Musashi mediates translational repression of the Drosophila hypoxia inducible factor — Musashi mediates translational repression of the Drosophila hypoxia inducible factor — SUPPLEMENTARY DATA 

# Musashi mediates translational repression of the *Drosophila* hypoxia inducible factor

## SUPPLEMENTARY DATA

- SUPPLEMENTARY DATA
