# Supplementary material for: Musashi mediates translational repression of the Drosophila hypoxia inducible factor
Source: Nucleic Acids Res. 2016 May 3;44(16):7555–67. doi: 10.1093/nar/gkw372 (PMC5027473; doi:10.1093/nar/gkw372)
Supplement: SUPPLEMENTARY DATA [file supp_gkw372_nar-03791-v-2015-File009.pdf]

SUPPLEMENTARY FIGURE LEGENDS

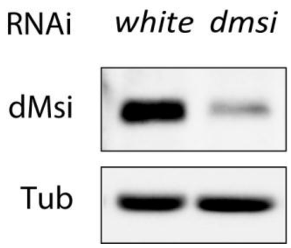

Suppl. Figure S1. Effective silencing of *dmsi* expression by interference RNA (RNAi) expressed in transgenic embryos. Western blot analysis showing reduced expression of dMsi protein in late-stage embryos in which actin-GAL4 promotes the expression of a UAS-*msi* RNAi transgene (VDRC#44895); the transgenic embryos also overexpress Dicer2 under control of the same Gal4 driver. Tubulin was used as a loading control.

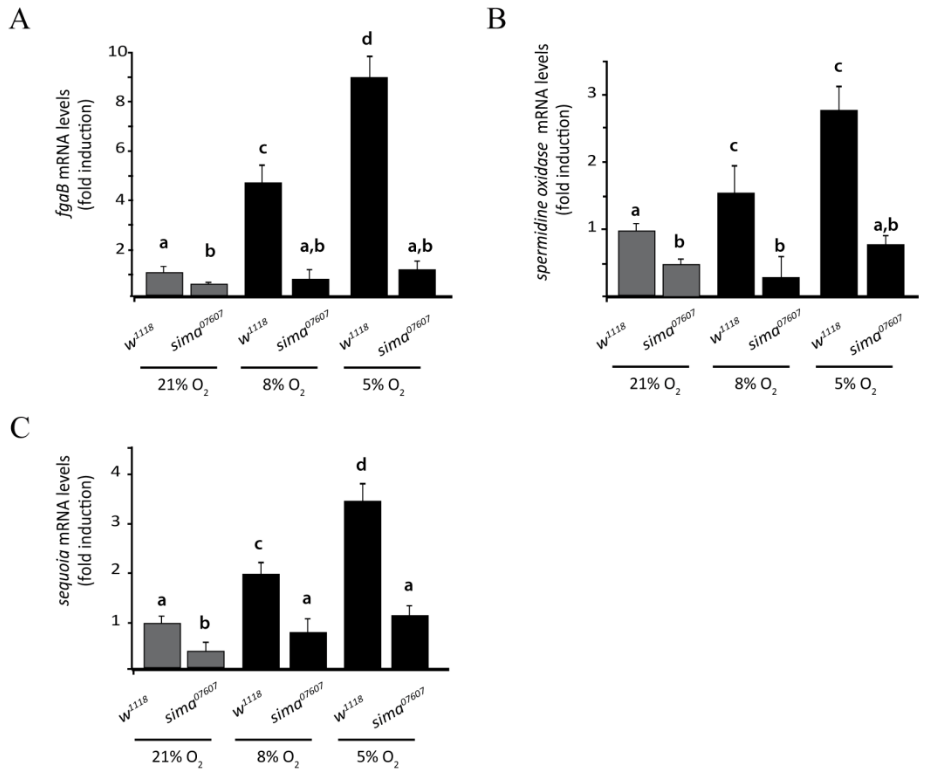

Suppl. Figure S2. Hypoxic induction of Sima target genes in 3rd instar larvae. *fga B* (A), *spermidine oxidase* (B) and *sequoia* (C) gene expression was analyzed by RT-qPCR in wild type or *sima*^{07607} mutant 3rd instar larvae maintained in normoxia, or exposed to mild (8% O_2 5h) or strong (5% O_2 5h) hypoxia. Both basal expression and hypoxic induction of all 3 hypoxia-inducible genes depend on Sima. Error bars represent SEM (n=3; different letters indicate statistical differences with a $p<0.05$ in a two-way ANOVA with a Bonferroni post-hoc test).

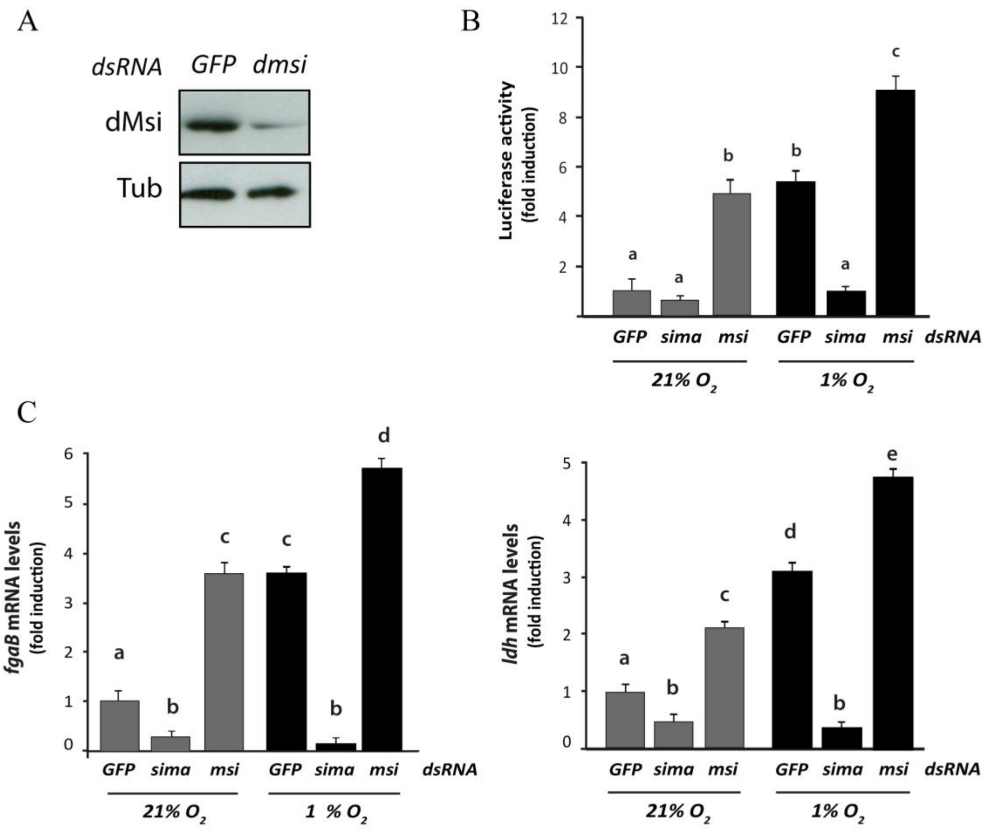

Suppl. Figure S3. Musashi represses Sima-dependent transcriptional responses in S2R+ cells. A) Western blot showing reduction of dMsi protein levels in cells treated with *dmsi* double stranded RNA during 48h. Tubulin was used as a loading control. B) Normoxic or hypoxic (1% O_2 20h) S2R+ cells stably transfected with a HIF-Responsive Element luciferase reporter (HRE-Luc) were treated with dsRNA against *dmsi*, using *GFP* or *sima* dsRNAs as controls. Silencing of *dmsi* provokes upregulation of HRE activity both in normoxia and hypoxia, in comparison with cells treated with the *GFP* dsRNA. In cells treated with sima dsRNA, luciferase induction in hypoxia is prevented. Luciferase activity is depicted as fold induction respect to cells treated with the *GFP* dsRNA maintained in normoxia. Note that oxygen levels routinely utilized to attain maximal HIF-dependent responses in S2R+ cells (1%O_2) are lower than those utilized in embryos (5% O_2), because in embryos HIF-dependent transcription is blocked at 1% O_2 (7). C) mRNA levels of two different Sima endogenous target genes, *fgaB* (left) and *ldh* (right), were analyzed by RT-qPCR in cells treated with *dmsi*, *GFP* or *sima* dsRNAs and then maintained in normoxia or exposed to hypoxia as in panel B). *dmsi* silencing leads to increased levels of *fgaB* and *ldh* expression both in normoxia and hypoxia. Rpl29 mRNA was used for normalization. Error bars represent SEM (n=3; different letters indicate statistical differences with a p<0.05 in a two-way ANOVA with a Bonferroni post-hoc test).

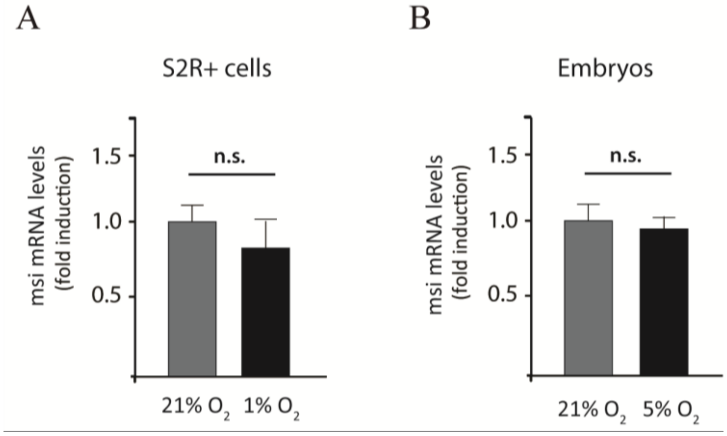

Suppl. Figure S4. dMsi mRNA levels are unaffected in hypoxia. *msi* mRNA levels were analyzed by RT-qPCR in S2R+ cells (A) or late-stage embryos (B) maintained in normoxia or exposed to hypoxia. *msi* mRNA levels are not affected by oxygen conditions. Rpl29 transcript levels were used to normalize quantifications. n.s., values not significantly different, Student's t-test.

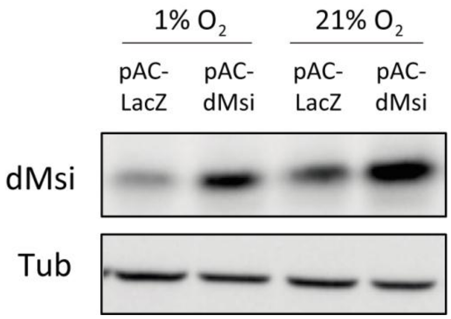

Suppl. Figure S5. Overexpression of dMsi in S2R+ cells overrides oxygen-dependent regulation of the protein. Western blot analysis showing dMsi levels in cells exposed to hypoxia (1% O_2 20h) or maintained in normoxia, which were transfected with a plasmid overexpressing dMsi in comparison with control cells expressing a LacZ construct. dMsi protein levels in hypoxic cells transfected with the dMsi overexpression plasmid are higher than levels in normoxic cells transfected with the control construct LacZ, indicating that overexpression of dMsi overrides the downregulation of the protein in hypoxia. Tubulin was used as a loading control.

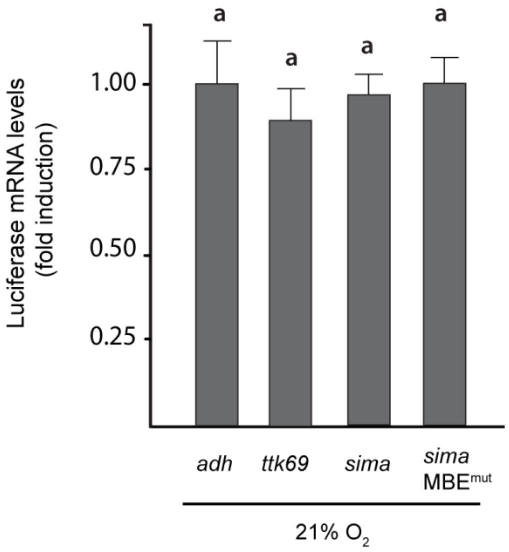

Suppl. Figure S6. *firefly* luciferase mRNA levels are unaffected by the presence of Musashi Binding Elements. S2R+ cells were transfected with luciferase reporters containing the *alcohol dehydrogenase* (*adh*) 3'UTR, *tramtrack69* (*ttk69*) 3'UTR, or *sima* 3'UTR (either wild type or mutated, MBE mut) and maintained in normoxia for 24h; mRNA levels of each of the transcripts was measured by RT-qPCR along with those of a *Renilla* luciferase reporter, which was co-transfected for normalization. Luciferase mRNA levels of all the transcripts are similar irrespective of the 3'UTR present in each construct. Error bars represent SEM (n=3; one way ANOVA; mean values with a letter in common are not significantly different).
